# Comparative Prevalence of Iron Deficiency Anemia in Exclusively Breastfed Versus Exclusively Cow Milk-Fed Infants: A Cross-Sectional Study

**DOI:** 10.7759/cureus.82916

**Published:** 2025-04-24

**Authors:** Faiza Gul, Srichand Mulakalapalli, Nida Gul, Ghulam Ishaq, Komal Ali, Ayaz Ali, Nadia Khan, Shah Faisal, Khayam, Mamoona

**Affiliations:** 1 Pediatrics, Lady Reading Hospital Peshawar, Peshawar, PAK; 2 Pediatrics, Dr. Pinnamaneni Siddhartha Institute of Medical Sciences, Vijayawada, IND; 3 Medicine, Medical Teaching Institution (MTI) Khyber Teaching Hospital, Peshawar, PAK; 4 Internal Medicine, Medical Teaching Institution (MTI) Khyber Teaching Hospital, Peshawar, PAK; 5 Internal Medicine, Combined Military Hospital, Peshawar, PAK; 6 Pediatrics, Women Medical and Dental College Abbottabad, Peshawar, PAK

**Keywords:** cross-sectional study, exclusively breast milk feed, exclusively cow milk feed, infants, iron deficiency anemia (ida)

## Abstract

Introduction: Anemia has long been a major public health issue worldwide and continues to be the most common type of micronutrient deficiency. Iron deficiency anemia (IDA) represents a major health challenge during infancy and early childhood. Children in Pakistan suffering from IDA often experience stunted growth, cognitive delays, and decreased physical activity.

Methodology: This cross-sectional study was conducted from October 2024 to February 2025 in the Pediatric Department of Lady Reading Hospital, Peshawar, to assess the prevalence of IDA in term infants up to 12 months of age who were either breastfed or fed cow's milk. Using non-probability purposive sampling, 340 eligible infants visiting the outpatient department or admitted to the pediatrics ward were included. Infants with hemoglobin <9.5 g/dL and mean corpuscular volume <70 fL were labeled as having IDA. Ethical approval and informed consent were obtained prior to data collection.

Results: ​In our study of 340 infants (mean age: 3.71 ± 1.84 months), 127 (37.4%) were diagnosed with IDA. A significant association was found between exclusive cow's milk feeding and IDA (p < 0.001), with 92 (72.44%) of IDA cases occurring in cow's milk-fed infants. No significant link was observed between the frequency of pediatric check-ups and IDA prevalence. Additionally, parental confidence in understanding their infant's nutritional needs was significantly related to IDA occurrence (p = 0.05).

Conclusion: The study's findings indicate a significant association between exclusive cow's milk feeding and the development of IDA in infants, underscoring the importance of appropriate infant feeding practices. No significant link was observed between the frequency of pediatric check-ups and IDA prevalence. Additionally, parental confidence in understanding their infant's nutritional needs was significantly related to IDA occurrence. These results highlight the need for targeted nutritional education for parents to prevent IDA in infants.

## Introduction

Iron deficiency anemia (IDA) has long been a major public health issue worldwide and continues to be the most common type of micronutrient deficiency, affecting an estimated 1.6 billion people across the globe in a data of 2012 [1]. It is due to its potential impact on cognitive development, immune function, and growth, which may lead to long-term developmental delays if left unaddressed.

IDA represents a major health challenge during infancy and early childhood [2]. Iron plays a vital role in various bodily functions, including the synthesis of hemoglobin, a crucial protein present in red blood cells. The body maintains iron reserves, but when these stores are exhausted, iron deficiency develops [3]. IDA arises when iron stores in the body are critically low, leading to reduced hemoglobin levels. It is typically marked by red blood cells that are smaller than normal and pale in color (microcytic and hypochromic) [4]. Although many infants with iron deficiency show no obvious symptoms, some may exhibit a range of issues, including difficulties with cognitive development and behavioral disturbances [5].

For the first six months of life, the nutritional requirements of healthy, full-term infants are generally fulfilled by breast milk, provided the mother maintains good nutritional status. However, in some cases, certain micronutrients may become insufficient before six months. Specifically for iron, the amount stored in the infant’s body at birth significantly influences the likelihood of developing anemia, since breast milk contains only small amounts of iron. Infants born at a normal weight to mothers with adequate iron levels during pregnancy typically have sufficient liver iron stores, which lowers the risk of iron deficiency in early infancy [6].

Infants who are fed cow's milk or formula are at a higher risk of developing iron deficiency. This is due to the low iron content in these milks, potential hidden intestinal blood loss linked to cow’s milk during infancy, and the reduced absorption of non-heme iron caused by components like calcium and casein. As children grow, they need to absorb about 0.5 mg more iron each day than they lose to sustain a healthy body iron level of around 200 mg [7,8].

Children in Pakistan suffering from IDA often experience stunted growth, cognitive delays, and decreased physical activity. These factors are also believed to play a role in the country's elevated infant mortality rate [9-12].

IDA is often considered a concern primarily in developing countries, and limited research has been conducted on assessing iron status in healthy infants who are breastfed [13].

This study aims to assess the prevalence of IDA among infants in Pakistan, where rising rates of IDA and limited awareness regarding optimal breastfeeding practices highlight the need for targeted investigation.

## Materials and methods

This cross-sectional study was conducted over a five-month period, from October to February 2025, in the Pediatric Department of Lady Reading Hospital, located in Peshawar, Pakistan. The study aimed to assess the prevalence of IDA among term infants up to 12 months of age who were either breastfed or consumed cow's milk. Preterm infants, individuals with chronic illnesses, and those currently receiving iron supplementation were excluded from the study.

Ethical approval was obtained from the Institutional Review Board of Lady Reading Hospital, ensuring that the study adhered to ethical research standards and protocols. Prior to data collection, informed consent was obtained from all participating parents or guardians to ensure voluntary participation and confidentiality of information.

The questionnaire was self-designed based on a comprehensive review of relevant literature and expert consultation to assess feeding practices, anemia history, and parental knowledge. It was reviewed by two pediatricians and a public health specialist for content validity. A pilot study was conducted on 34 parents from the same hospital population to assess clarity, comprehension, and timing. Minor revisions were made based on feedback.

The data collection utilized a non-probability purposive sampling technique. This approach was chosen to specifically target infants up to 12 months who visited the Outpatient Department (OPD) of Lady Reading Hospital during the study period. These infants were included in the study if they met the criteria of being exclusively breastfed or fed cow's milk. After obtaining informed consent, blood samples were collected from the children, and a complete blood count (CBC) was performed to identify IDA. Infants having hemoglobin <9.5 g/dl and mean corpuscular volume (MCV) <70 fL were labeled as having IDA. Infants who visited the pediatrics OPD or were admitted to the pediatric ward at Lady Reading Hospital and met the inclusion criteria were included in the study. A total of 340 patients were included in this study.

Data analysis

Data were analyzed using SPSS Statistics software, version 22 (IBM Corp., Armonk, NY). The analysis focused on exploring the association between IDA and the type of feeding, either exclusive cow's milk or exclusive breastfeeding, among the infants included in the study.

The chi-square test of independence was applied to determine whether there was a statistically significant association between the incidence of IDA and the type of milk consumption. For the purposes of this study, a p-value of less than 0.05 was considered statistically significant. Conversely, p-values greater than 0.05 were considered insignificant, suggesting no meaningful association between the feeding method and the presence of IDA in the infants studied.

The results of the chi-square test were used to evaluate potential differences in the prevalence of IDA between breastfed infants and those consuming cow's milk.

## Results

In this study, a total of 340 infants participated, with a gender distribution of 126 males (37.1%) and 214 females (62.9%). The mean age of the participants was 3.71 months, with a standard deviation of ±1.842 months. Regarding feeding practices, 196 (57.6%) infants were exclusively breastfed, and 144 (42.4%) infants were exclusively fed cow's milk. In our study, 127 infants (37.4%) were diagnosed with IDA. Among those diagnosed with IDA, 92 infants (72.44%) were exclusively fed cow's milk.

We applied the chi-square test to find the association between cow milk feeding and the development of IDA, and it showed a significant prevalence of <0.001, indicating a strong association between exclusive cow's milk feeding and the development of IDA in infants.

This significant association suggests that infants who are exclusively fed cow's milk are at a higher risk of developing iron deficiency anemia compared to those who are breastfed. These findings underscore the importance of considering the nutritional sources for infants and the potential impact on their health, particularly concerning iron intake and deficiency.

Parents of the infants were surveyed about the frequency of regular pediatric check-ups for their children. Among the respondents, 142 parents (41.76%) reported that they conducted regular check-ups for their infants, 108 parents (31.76%) indicated they conducted occasional check-ups, and 90 parents (26.47%) reported that they did not conduct regular check-ups.

No significant association was found between pediatric check-ups and IDA, indicating that the frequency of pediatric check-ups did not have a statistically significant impact on the likelihood of infants developing IDA. The details are given in Table 1.

**Table 1:** Factors associated with iron deficiency anemia. The chi-square test is significant when p < 0.05. Each percentage was calculated based on the total number of patients (340).

Variables		Iron deficiency anemia		P-value	Chi-square value
		Yes, n (%)	No, n (%)		
Feeding method	Breastfeed	35 (10.29)	161 (47.35)	<0.001	75.167
	Cow’s milk	92 (27.05)	52 (15.29)		
Does the infant have regular pediatric check-ups?	Yes, regularly	50 (14.71)	92 (27.05)	0.532	1.261
	Yes, occasionally	45 (13.23)	63 (18.52)		
	No	32 (9.41)	58 (17.05)		
How confident do you feel about your understanding of the nutritional needs of your infant?	Very confident	2 (0.58)	16 (4.70)	0.05	5.762
	Somewhat confident	44 (12.94)	65 (19.11)		
	Not confident	81 (23.82)	132 (38.82)		

Out of 345 participants, 232 parents (67.2%) reported a lack of confidence in understanding the nutritional needs of their infants. Among the 89 non-anemic infants, 58 parents (65.2%) also reported low confidence in this area. The association was statistically significant with a p-value of 0.05.

## Discussion

IDA remains the most prevalent and treatable form of anemia globally. Once the underlying cause, such as blood loss or dietary deficiency, is identified, the diagnosis of anemia can be confirmed through the presence of microcytic, hypochromic red blood cells along with abnormal serum biochemical markers, including low ferritin, low iron levels, and reduced transferrin saturation [14]. Clinical attention is primarily directed toward the early detection of subclinical iron deficiency to prevent potential systemic complications and the identification of IDA, which can result from insufficient dietary iron, impaired iron absorption, or excessive blood loss. This form of anemia typically responds well to appropriate treatment [15].

Iron deficiency has been linked to rapid growth and exclusive breastfeeding beyond six months during the first year of life, as well as the introduction of regular (non-fortified) cow’s milk before the age of 12 months. However, the overall length of breastfeeding has shown a positive correlation with iron levels [16-18].

In our study, we included 340 infants aged from birth to 12 months. The findings revealed that a larger proportion of the infants in our sample were female. Additionally, a significant number of anemic infants were predominantly fed cow's milk. This suggests that feeding practices, particularly the use of cow's milk, may be associated with a higher risk of anemia in this age group. The low iron content in cow's milk, along with potential issues with iron absorption, may contribute to the development of IDA. These results are consistent with findings from another study, which reported a higher prevalence of IDA among infants on cow's milk compared to those exclusively breastfed (37.7% vs. 22.2%) [19]. A meta-analysis conducted found that the evidence supporting an increased risk of anemia associated with cow's milk, compared to formula milk, was of low certainty [20]. Another study shows that the incidence of infectious disease was much lower in those who were breastfed as compared to those who were fed on formula milk or cow milk [21]. This difference may be attributed to the fact that cow's milk contains relatively low bioavailable iron and can also interfere with iron absorption. In contrast, breast milk provides a more balanced source of nutrients, including higher bioavailability of iron, which is crucial for proper growth and development during infancy. Therefore, our findings highlight the importance of promoting exclusive breastfeeding in the first year of life as a strategy to reduce the risk of IDA.

In our study, parents were asked about the regularity of pediatric check-ups for their infants. The majority of parents reported that they did not schedule regular pediatric visits for their children. A significant association was found between the lack of regular pediatric check-ups and the prevalence of anemia. This finding suggests that the absence of routine healthcare visits may contribute to the delayed detection and management of conditions like IDA. Regular pediatric check-ups play a crucial role in monitoring the growth, development, and nutritional status of infants. These visits offer an opportunity for early identification of nutritional deficiencies and other health issues, allowing for timely intervention. The lack of such visits could potentially lead to undiagnosed anemia and other preventable health complications in this vulnerable age group.

Limitations

The limitations of this study include its cross-sectional design, which restricts the ability to infer causal relationships. Additionally, the study was conducted at a single center, limiting the generalizability of the findings. Furthermore, a non-probability sampling technique was employed, which may introduce selection bias.

## Conclusions

The study's findings underscore the significant impact of feeding practices on infants' iron status. Specifically, exclusive cow's milk feeding was strongly associated with a higher incidence of IDA, with 72.44% of IDA cases occurring in infants exclusively fed cow's milk. This aligns with existing research indicating that cow's milk has low iron content and can inhibit iron absorption, contributing to anemia in young children. In contrast, the frequency of pediatric check-ups did not show a significant correlation with IDA prevalence, suggesting that while regular medical visits are vital for overall health monitoring, they may not directly influence iron status.​

Additionally, a significant relationship was observed between parental confidence in understanding their infant's nutritional needs and the occurrence of IDA. This highlights the importance of enhancing parental education on proper infant nutrition to prevent iron deficiency.​ In summary, these results emphasize the critical role of appropriate dietary practices and the need for targeted nutritional education for parents to mitigate the risk of IDA in infants.

## Appendices

Questionnaire

Instructions

Please answer the questions honestly. If you answer "Yes" to any one of these questions, 3, 4, 5, 6, 7, and 8, you may skip the remaining questions.

Section A: Infant Demographics

1. Infant’s age (in months): _______

2. Infant’s gender:

Male

Female

Section B: Iron Deficiency Screening

3. Has your infant ever been diagnosed with iron deficiency anemia?

Yes ⟶ (If Yes, please stop here. Thank you for your participation.)

No

Don’t know

4. Was your child born premature/preterm?

Yes

No

5. Is your child currently using iron supplements?

Yes

No

6. Does your child have any chronic infection?

Yes

No

7. Is your child malnourished?

Yes

No

8. Are you giving your child any source of nutrition other than exclusive breastfeeding or exclusive cow’s milk?

Yes

No

Section C: Feeding Practices

9. What is your infant’s current feeding method?

Exclusively breastfed

Exclusively fed cow’s milk

Section D: Parental Knowledge and Healthcare Behavior

10. How confident do you feel about understanding the nutritional needs of your infant?

Very confident

Somewhat confident

Not confident

11. Does the infant have regular pediatric check-ups?

Yes, regularly

Occasionally

No
